# Identification and analysis of novel small molecule inhibitors of RNase E: Implications for antibacterial targeting and regulation of RNase E

**DOI:** 10.1016/j.bbrep.2020.100773

**Published:** 2020-06-09

**Authors:** Charlotte E. Mardle, Layla R. Goddard, Bailei C. Spelman, Helen S. Atkins, Louise E. Butt, Paul A. Cox, Darren M. Gowers, Helen A. Vincent, Anastasia J. Callaghan

**Affiliations:** a School of Biological Sciences and Institute of Biological and Biomedical Sciences, University of Portsmouth, Portsmouth, PO1 2DY, United Kingdom; b Defence Science and Technology Laboratory, Porton Down, Salisbury, United Kingdom; c University of Exeter, Exeter, United Kingdom; d London School of Hygiene and Tropical Medicine, London, United Kingdom; e School of Pharmacy and Biomedical Sciences and Institute of Biological and Biomedical Sciences, University of Portsmouth, Portsmouth, PO1 2DT, United Kingdom

**Keywords:** Antibacterial target, Endoribonuclease RNase E, Metabolite-mediated regulation, Small molecule inhibitor, Virtual high-throughput screening (vHTS)

## Abstract

Increasing resistance of bacteria to antibiotics is a serious global challenge and there is a need to unlock the potential of novel antibacterial targets. One such target is the essential prokaryotic endoribonuclease RNase E. Using a combination of *in silico* high-throughput screening and *in vitro* validation we have identified three novel small molecule inhibitors of RNase E that are active against RNase E from *Escherichia coli*, *Francisella tularensis* and *Acinetobacter baumannii*. Two of the inhibitors are non-natural small molecules that could be suitable as lead compounds for the development of broad-spectrum antibiotics targeting RNase E. The third small molecule inhibitor is glucosamine-6-phosphate, a precursor of bacterial cell envelope peptidoglycans and lipopolysaccharides, hinting at a novel metabolite-mediated mechanism of regulation of RNase E.

## Highlights

• RNase E, an essential bacterial endoribonuclease, is a potential antibacterial target.
• Three novel small molecule inhibitors of RNase E are identified.
• Each inhibitor is active against RNase E from *E. coli*, *F. tularensis* and *A. baumannii*.
• Two, as non-natural compounds, are suitable lead compounds for antibiotic development.
• One, a metabolite, is a potential novel regulator of RNase E.

## 1 Introduction

For the majority of bacterial infections, the only treatment(s) available are antibiotics. However, many traditional antibiotics are now ineffective due to the emergence of antimicrobial resistant strains and, consequently, there is a growing need for the development of novel antibacterial strategies. RNA degradation has recently been identified as a cellular process that could potentially be targeted by antibiotics [1,2]. This is because RNA degradation is both an essential component of RNA metabolism and there are fundamental differences between prokaryotic and human ribonucleases (RNases), the enzymes responsible for RNA turnover (reviewed in Refs. [1,2]). One RNase has been specifically identified as a potential antibacterial target, the endoribonuclease RNase E [1].

RNase E is an attractive antibacterial target for several reasons. Firstly, RNase E is an essential enzyme, meaning that RNase E inhibitors would be expected to have antibacterial properties. Studies conducted in the late 1970s demonstrated that inactivation of RNase E is lethal to *Escherichia coli* [3,4]. Since then, considerable effort has been expended in trying to understand the role(s) that RNase E plays. In *E. coli*, RNase E is now known to be multi-functional, having critical roles in rRNA and tRNA maturation and mRNA and rRNA decay (see Refs. [5,6] for recent reviews of RNase E functions). It is possible that RNase E is indispensable as a consequence of this broad functionality. However, more recent studies are beginning to suggest that there may be very specific functions of RNase E that are necessary under certain growth conditions [7,8]. A second reason for selecting RNase E as an antibacterial target is that RNase E has been reported to play direct roles in the virulence of the pathogens *Salmonella enterica* [9] and *Yersinia pestis* [10]. Suppression of virulence phenotypes generally does not kill bacteria, but it does disarm them, and targeting virulence factors has been identified as a valid platform for developing antibiotics [reviewed in 11]. Finally, RNase E is a suitable antibacterial target because it is highly conserved and widely distributed amongst Gram-negative bacteria but there is no known orthologue in humans [12]. Therefore, it would be hoped that specific RNase E inhibitors would be detrimental to bacterial survival but non-toxic to humans.

RNase E endoribonucleolytically cleaves within single-stranded A/U-rich regions of its RNA substrates and has a strong preference for substrates with a 5′ monophosphate [13,14]. The N-terminal domain (NTD) of RNase E is responsible for the endoribonuclease activity [15]. It is a homotetramer, organised as a dimer of dimers, with each monomeric unit consisting of five subdomains: an RNase H domain, an S1 domain, a 5′ sensor domain, a deoxyribonuclease (DNase) I domain and a small domain [16]. The active site is formed by the DNase I and S1 subdomains [16]. It contains an essential catalytic magnesium ion, coordinated by two aspartates from the DNase I subdomain (D303, positioned by N305, and D346 in *E. coli* RNase E), that is required for hydrolytic cleavage of an RNA substrate and an RNA-binding site, the uracil pocket of the S1 subdomain (including key amino acids F57, F67 and K112), that determines the A/U-rich substrate specificity [16,17] (Supplementary Fig. S1). The phosphorylation state of the substrate is recognised by the 5′ sensor subdomain through interactions between a 5′ monophosphate and conserved arginine and threonine residues (R169 and T170 in *E. coli* RNase E) which are positioned by a conserved glycine and valine (G124 and V128 in *E. coli* RNase E) [16,18,19] (Supplementary Fig. S1). We reasoned that any small molecule capable of binding at, and therefore blocking, the active site and/or the 5′ sensor region would be a potential inhibitor of RNase E [20].

As a first step in realising the potential of RNase E as an antibacterial target, a recent collaboration between our lab and the McDowall group (University of Leeds, UK) used structure-based virtual high-throughput screening (vHTS) against the active site and 5′ sensor region of *E. coli* RNase E to identify the first small molecule inhibitors of RNase E [20]. Unfortunately, the inhibitors identified in Kime et al. [20] are no longer commercially available at a cost that would enable us to explore their development as antimicrobials. Therefore, we decided to search for inhibitors that are commercially available and, ideally, are relatively inexpensive. In the current study we have now identified and characterised a further three novel small molecule inhibitors of RNase E, all of which are commercially available and inexpensive. Initially, structure-based vHTS was performed, using a screening library of commercially available chemical building blocks, to identify small molecules predicted to inhibit RNase E by binding to/blocking the active site or 5′ sensor region. Candidate inhibitors were then filtered by docking score, known physicochemical properties and economic factors; resulting in the selection of eleven small molecules that were screened *in vitro* for inhibitory activity against purified *E. coli* RNase E NTD. The small molecules that inhibited *E. coli* RNase E NTD were: AS2, a non-natural small molecule, predicted to target the active site; AS4/glucosamine-6-phosphate (GlucN6P), a natural precursor of bacterial cell envelope peptidoglycans and lipopolysaccharides, also predicted to target the active site; and 5′S1, a non-natural small molecule, predicted to target the RNA-binding 5′ sensor region. Furthermore, each inhibitor also inhibited the RNase E NTD from bacterial pathogens of importance to the health (*Acinetobacter baumannii* [21]) and defence (*Francisella tularensis* [22]) sectors. We anticipate that the identified novel small molecule RNase E inhibitors will provide a foundation for the development of broad-spectrum antibiotics targeting RNase E. In addition, the finding that RNase E is inhibited by the metabolite GlucN6P suggests that RNase E activity could be regulated via a metabolite-mediated mechanism.

## 2 Materials and Methods

### 2.1 Structure-based virtual high-throughput screening (vHTS) for small molecule inhibitors of RNase E

*RNase E structure preparation* – *E. coli* RNase E NTD crystal structures (closed conformation: 2BX2, [16]; open conformation: 2VMK [18]) were opened in the program MOE (Molecular Operating Environment, 2013.08; Chemical Computing Group Inc., 1010 Sherbrooke St. West, Suite #910, Montreal, QC, Canada, H3A 2R7). An apo-2BX2 structure was generating by removing the co-crystallised bound RNA substrate from 2BX2. MOE's QuickPrep function was used to subject apo-2BX2 and 2VMK to protonation and energy minimisation, using the Amber12:EHT force field parameters [23,24].

*Identification of putative small molecule-binding sites in RNase E* – The MOE Alpha Site Finder function was used to identify putative small molecule-binding sites in the prepared *E. coli* RNase E NTD structures. A putative small molecule-binding site at the active site of *E. coli* RNase E was selected in the apo-2BX2 structure based on the presence of the catalytic residues D303, N305 and D346 and a putative small molecule-binding site at the 5′ sensor region was selected in the 2VMK structure based on the presence of the key amino acids G124, V128, R169 and T170. Each putative small molecule-binding site was defined by the placement of dummy atoms.

*Small molecule structure preparation* – Structures of P6 (Maybridge: HTS01081) and P11 (Maybridge: SEW06445) were retrieved from Maybridge [25] and the structures for the Sigma Aldrich (Building Blocks) screening library (67,449 compounds from a vendor catalogue of 109,823 compounds) were retrieved from the ZINC database [26,27]. All small molecule structures were prepared using the QuickPrep function in MOE as described above for the RNase E structures.

*In silico molecular docking of small molecules into the active site of RNase E* – The MOE Dock function was used to dock the small molecules from the Sigma Aldrich (Building Blocks) screening library into the active site of the apo-2BX2 *E. coli* RNase E NTD structure using ‘Triangle Matcher’ placement methodology and 30 placement poses. RNase E-small molecule complexes were scored according to the London dG scoring function (MOE) and subjected to a rigid receptor refinement step with no second rescoring. The 10 lowest-energy unique RNase E-small molecule complex conformations were retained for each small molecule.

*In silico molecular docking of small molecules into the 5*′ *sensor region of RNase E* – The MOE Dock function was used to dock RNase E inhibitors P6 (Maybridge: HTS01081) and P11 (Maybridge: SEW06445) [20] into the 5′ sensor region of the 2VMK *E. coli* RNase E NTD structure as described above for docking small molecules into the active site. The results were combined to generate a pharmacophore query in MOE. This was applied to the small molecules from the Sigma Aldrich (Building Blocks) screening library. 500 small molecules with the lowest root-mean-square deviation (RMSD) compared to the pharmacophore query were redocked into the 5′ sensor of the 2VMK *E. coli* RNase E NTD structure using the docking parameters described above for docking small molecules into the active site.

*Filtering of the vHTS results* – The vHTS results against the active site and the 5′ sensor region of *E. coli* RNase E were collated and were ranked from best to worst based on docking score. Small molecules with known undesirable physicochemical properties (nucleosides, nucleotides and nucleoside/nucleotide analogues; metal chelators; insoluble small molecules; unstable small molecules; toxic small molecules) were removed. Finally, small molecules reported to have desirable bioactivity were masked before removing small molecules that cost more than £5 per mg.

### 2.2 Molecular docking of selected small molecules into *E. coli* RNase E NTD

Following vHTS, the Dock function in MOE was used to dock the selected small molecules into the apo-2BX2 *E. coli* RNase E NTD structure. Small molecules AS1-9 were docked into the active site and small molecules 5′S1 and 5′S2 into the 5′ sensor region of apo-2BX2 using ‘Triangle Matcher’ placement methodology and 100 placement poses. RNase E-small molecule complexes were scored according to the London dG scoring function (MOE) and subjected to a rigid receptor refinement step with no second rescoring. The 30 lowest-energy unique RNase E-small molecule complex conformations were retained for each small molecule.

### 2.3 Cloning, expression and purification of RNase E NTDs

Cloning, expression and purification of the NTDs from *E. coli* RNase E (aa 1–529), *F. tularensis* RNase E (aa 1–543) and *A. baumannii* RNase E (aa 1–544) has been described previously [28]. Briefly, each of the RNase E NTDs was expressed as an N-terminally His_10_-tagged protein from the pET16b expression vector (Novagen). The expression vector for *E. coli* RNase E NTD was kindly provided by Prof. Ben Luisi (University of Cambridge, UK). Codon-optimised genes for *F. tularensis and A. baumannii* RNase E NTD were obtained from GeneArt (Life Technologies) and ligated between the *Nde*I and *Bam*HI restriction sites of pET16b. Each RNase E NTD was expressed in *E. coli* BL21(DE3)pLysS transformed with the respective expression vector. Each expression strain was grown to OD_600_ = 0.6 in 500 ml LB supplemented with 100 μg/ml ampicillin, at 37 °C, with shaking at 250 rpm. Isopropyl β-d-1-thiogalactopyranoside (IPTG) was added to a final concentration of 1 mM to induce expression of the RNase E NTD. Cells were incubated for a further 3 h, at 37 °C, with shaking at 250 rpm before being harvested by centrifugation at 7000 rcf and 4 °C for 20 min. The cell pellet was stored frozen at -20 °C prior to RNase E NTD purification. Frozen cell pellets were thawed on ice and resuspended in 50 ml Buffer A (20 mM Tris-HCl (pH 8), 500 mM NaCl, 20 mM imidazole) supplemented with a cOmplete^TM^ ethylenediaminetetraacetic acid (EDTA)-free protease inhibitor cocktail tablet (Roche). Cells were lysed by sonication (Sonics Vibra Cell VCX 500 sonicator): 3.3 s on, 9.9 s off for 10 min. Lysate was clarified by centrifugation at 40,000 g and 4 °C for 20 min and loaded onto a 5 ml HisTrap FF column (GE Healthcare) equilibrated in Buffer A using an ÄKTA Purifier (GE Healthcare). Bound proteins were eluted in a linear gradient to 100% Buffer B (20 mM Tris-HCl (pH 8), 500 mM NaCl, 500 mM imidazole) applied over 30 ml. Fractions containing RNase E NTD were pooled and loaded onto a HiPrep 26/10 Desalting column (GE Healthcare) equilibrated in Buffer C (20 mM Tris-HCl (pH 8), 500 mM NaCl, 10 mM MgCl_2_, 10 mM EDTA, 10 mM dithiothreitol (DTT) and 10% (v/v) glycerol) using an ÄKTA Purifier. Fractions containing RNase E NTD were pooled, concentrated by centrifugation using a Vivaspin 20 (MWCO 10 kDa) centrifugal concentrator (Sartorius) and stored frozen at -80 °C.

### 2.4 *In vitro* screening of candidate small molecule inhibitors against *E. coli* RNase E NTD (discontinuous RNase E assay)

Discontinuous RNase E assays were carried out essentially as described previously [28]. Briefly, control reaction mixtures (30 μl) contained: 5 nM purified *E. coli* RNase E NTD; 1 μM 5′ phosphorylated, 3′ fluorescein amidite (FAM)-labelled p-UUUACAGUAUUUG-FAM (5′-p-RNA13-FAM-3′) RNA substrate (Dharmacon); 25 mM Tris-HCl (pH 8); 100 mM NaCl; 15 mM MgCl_2_; 1 mM DTT; 37.5 mg/ml Ficoll 70; and 5% (v/v) dimethyl sulfoxide (DMSO). Screening reaction mixtures were identical to the control except that they were supplemented with 0.625, 1.25, 2.5, 5 or 10 mM of the respective small molecule (Sigma), as indicated. All reactions were incubated at 28 °C for 45 min and terminated by the addition of 0.5 vol of quench buffer (95% (v/v) formamide, 20 mM EDTA). The reaction products were resolved by denaturing 7.5 M urea 20% polyacrylamide gel electrophoresis (PAGE) and gels were visualised using a G:Box UV transilluminator (Syngene).

### 2.5 Real-time FRET-based RNase E assay

Assays were carried out in black 96-well microplates (ThermoFisher Scientific). Modified target-guide RNA substrate was prepared by annealing a 5′ hydroxylated, 3′ FAM-labelled 18-mer (5′–OH–GGAUCGGAGUUUUAAAUU-FAM-3′) target RNA (Dharmacon) and a 5′ phosphorylated, 3′ tetramethyl-6-carboxyrhodamine (TAMRA)-labelled 13-mer (5′-p-UUUUCUCCGAUCC-TAMRA-3′) guide RNA (Dharmacon) at a molar ratio of 1:1.14 at 28 °C for 15 min. Reaction mixtures (100 μl) contained: 5 nM RNase E NTD; 0.1–2 μM modified target-guide RNA substrate; 0–10 mM AS2, AS4 or 5′S1; 25 mM Tris-HCl (pH 8); 100 mM NaCl; 15 mM MgCl_2_; 1 mM DTT; 37.5 mg/ml Ficoll 70; 5% (v/v) DMSO. Reaction components were incubated at 28 °C for 15 min prior to initiating the reaction by the addition of the modified target-guide RNA substrate. Fluorescence intensity measurements were collected at 32-s intervals for 2 h using a BioTek H1 Synergy plate reader with excitation and emission wavelengths of 494 nm and 520 nm, respectively. Data corresponding to up to 50% reaction completion were used to determine the rate of RNA cleavage.

To determine the half maximal inhibitory concentration (IC_50_), data were fitted to a three-parameter IC_50_ single-site inhibition model in GraFit5 (Erithacus software):

$$y=\frac{Range}{1+{\left(\frac{x}{{\text{IC}}_{50}}\right)}^{s}}$$

where *y* is the rate of RNA cleavage determined at inhibitor concentration *x*; *Range* is the difference between the theoretical maximal and minimal rate of RNA cleavage; IC_50_ is the concentration of inhibitor at half the *Range*; and *s* is the slope factor (a measure of how steeply the linear portion of the sigmoid falls).

For kinetic analysis, data were fitted to the Michaelis-Menten equation in GraFit5:

$$V_{0}=\frac{V_{max}\times \left[S\right]}{\left(K_{m}+\left[S\right]\right)}$$

where *V*_*0*_ is the measured rate of RNA cleavage, *V*_*max*_ is the maximal theoretical velocity, [*S*] is the concentration of RNA substrate and *K*_*m*_ is the apparent Michaelis constant.

## 3 Results

### 3.1 Structure-based vHTS for small molecule inhibitors of RNase E

In order to identify candidate RNase E inhibitors with the potential to bind at the active site and/or 5′ sensor region of RNase E we decided to use structure-based vHTS. As part of a collaborative project, we previously identified the first small molecule inhibitors of RNase E through structure-based vHTS of a Maybridge screening library against the active site and 5′ sensor region of *E. coli* RNase E [20]. A potential benefit of using a Maybridge screening library for vHTS is that it may increase the chance of identified inhibitors being promising lead compounds for drug development because the screening libraries have been specifically designed to contain chemical building blocks (small molecules that can be readily converted to secondary chemicals and intermediates) with drug-like properties [25]. However, Maybridge use Lipinski's rule of five (≤ 5 hydrogen bond donors, ≤ 10 hydrogen bond acceptors, molecular mass ≤ 500 Da and an octanol-water partition coefficient (log *P*) ≤ 5) [29] to assign drug-like properties. While these rules have proved useful for predicting which small molecules can penetrate human cells, they are less useful for predicting small molecule-penetration of bacterial cells, a prerequisite for the majority of antimicrobials [11]. Also, the commercial availability of the small molecules included in the Maybridge screening libraries is variable. Unfortunately, the monetary cost of obtaining the “Maybridge” RNase E inhibitors identified in Kime et al. [20], in the amounts required for further analysis, was prohibitive. Therefore, we decided to conduct a second vHTS, this time using a screening library of commercially available small molecules. To this end, we selected the Sigma Aldrich (Building Blocks) screening library that is available through the ZINC database because it is composed of chemical building blocks (small molecules with potential for chemical elaboration) that are commercially available from Sigma Aldrich [26,27]. The Sigma Aldrich (Building Blocks) screening library includes 67,449 small molecules from a catalogue of 109,823 [26]. Although ZINC provide an option to further filter the small molecules included in the screening library based on their perceived drug-like properties, due to the questionable relevance of applying Lipinski's rule of five to antimicrobials, we did not do so.

The first round of structured-based vHTS focused on identifying small molecules with the potential to bind at the active site of RNase E. A putative ligand-binding pocket at the active site of *E. coli* RNase E was defined based on the presence of amino acids D303, N305 and D346 (see Materials and Methods for details). The complete Sigma Aldrich (Building Blocks) screening library (67,449 compounds) was then screened against this site. A second round of vHTS targeted the 5′ sensor region. A potential ligand-binding site at the 5′ sensor region was defined based on the presence of amino acids G124, V128, R169 and T170 (see Materials and Methods for details). Due to the higher incidence of false positives for small molecules predicted to bind at the 5′ sensor region (18/21) compared to the active site (6/9) during our previous vHTS [20], we decided to prefilter the Sigma Aldrich (Building Blocks) screening library using a pharmacophore query based on previously reported RNase E inhibitors P6 and P11 [20] (see Materials and Methods for details). A subset of 500 small molecules (those with the lowest RMSD relative to the pharmacophore query) were subsequently screened against the 5′ sensor region of *E. coli* RNase E.

The results from both rounds of vHTS were collated and the small molecules were ordered from best to worst based on docking score. The results were then filtered to remove small molecules with undesirable physicochemical properties. This included the removal of nucleosides, nucleotides and nucleoside/nucleotide analogues; metal chelators e.g. EDTA; small molecules known to be insoluble; small molecules known to be unstable; and small molecules known to be toxic. These results were then masked to ensure the inclusion of small molecules with reported desirable bioactivity before filtering the remaining results to remove small molecules that were not commercially available at a reasonable cost using an arbitrary monetary cutoff of £5 per mg. Masking small molecules with desirable bioactivity ensured the inclusion of heparin, which does not meet the monetary cutoff but has been reported to have antimicrobial properties [30]. Using these criteria, nine small molecules predicted to bind/block the active site (AS1-9) and two small molecules predicted to bind/block the 5′ sensor region (5′S1 and 5′S2) were selected for further analysis (see Supplementary Table S1 for molecular details).

**Fig. 1:**
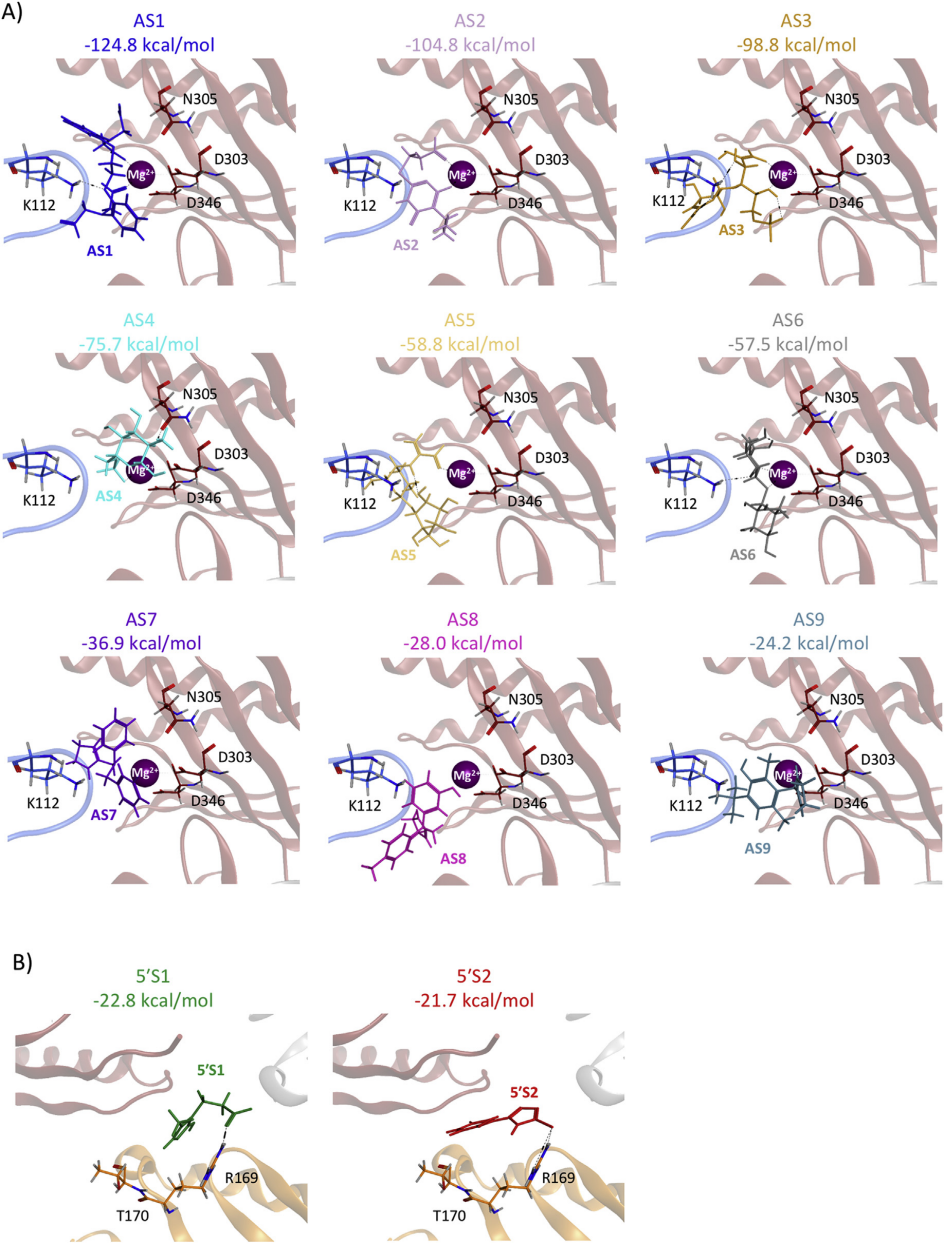
**Molecular docking of potential small molecule inhibitors into *E. coli* RNase E NTD.** The lowest-energy RNase E-small molecule complex conformations obtained from the molecular docking of potential small molecule inhibitors AS1-9 into the active site (A) and 5′S1 and 5′S2 into the 5′ sensor region (B) of *E. coli* RNase E NTD using 100 starting placement poses. The corresponding docking score is shown above each panel. The docked small molecule is shown as sticks and labelled in each panel. *E. coli* RNase E NTD is shown as a ribbon representation (S1 subdomain, blue; DNase I subdomain, red; 5′ sensor subdomain, gold; small subdomain, grey). Key amino acids, required for catalytic activity and/or substrate binding, are shown as sticks and labelled. (For interpretation of the references to colour in this figure legend, the reader is referred to the Web version of this article.)

These small molecules were redocked into *E. coli* RNase E using a similar process to the vHTS. However, a greater number of starting placement poses were used (100 compared to 30) and a greater number of unique RNase E-small molecule complex conformations were retained (30 compared to 10) to increase the likelihood of finding the lowest-energy RNase E-small molecule complex conformation. The lowest-energy RNase E-small molecule complex for each of the potential small molecule inhibitors docked into the active site (small molecules AS1-9) or the 5′ sensor region (small molecules 5′S1 and 5′S2) of *E. coli* RNase E, together with the corresponding docking score, is shown in Fig. 1.

### 3.2 *In vitro* screening of candidate small molecule inhibitors against *E. coli* RNase E NTD

**Fig. 2:**
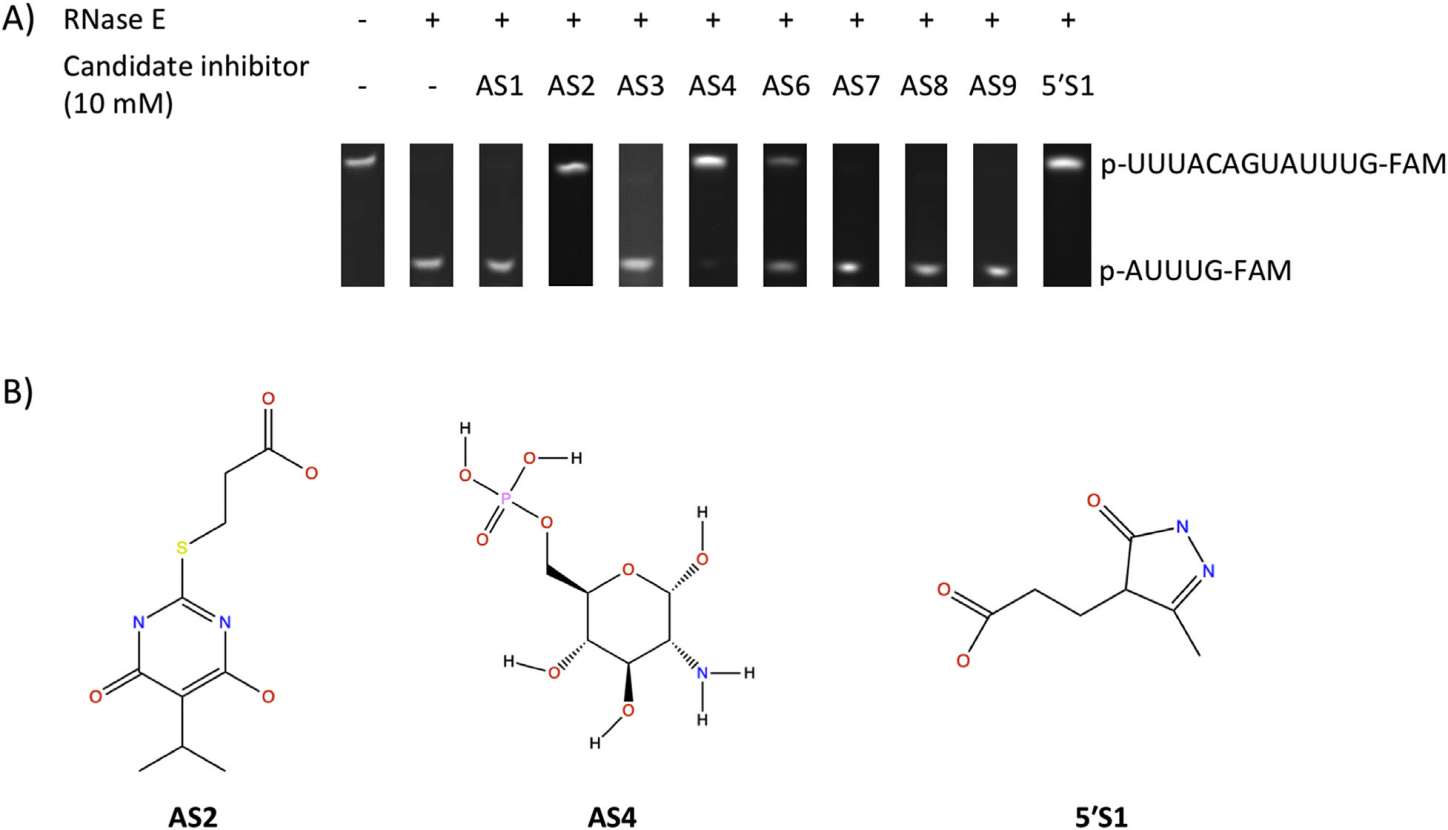
***In vitro* screening of candidate small molecule inhibitors against *E. coli* RNase E NTD.** (A) Representative 20% denaturing PAGE analysis of the cleavage of 1 μM 5′-p-RNA13-FAM-3′ by 5 nM E*. coli* RNase E NTD after incubation at 28 °C for 45 min in the absence of small molecule (-) or in the presence of 10 mM AS1, AS2, AS3, AS4, AS6, AS7, AS8, AS9, or 5′S1. This is a composite image assembled from multiple gels (complete gels are presented in Supplementary Fig. S2). The expected position of the bands representing the full-length FAM-labelled 5′-p-RNA13-FAM-3′ RNA substrate and the FAM-labelled pentamer 5′-p-AUUUG-FAM-3′ cleavage product are indicated on the right-hand-side of the gels. (B) The chemical structures of inhibitory small molecules AS2, AS4 and 5′S1 (further details are presented in Supplementary Table S1).

In order to determine whether the small molecules selected through vHTS inhibit RNase E, AS1-9, 5′S1 and 5′S2 were screened for inhibitory activity against *E. coli* RNase E NTD using an *in vitro* discontinuous assay that has been described previously [28]. In this assay, 5′-p-RNA13-FAM-3′, a 13-mer model RNA substrate of RNase E [16,31], is cleaved by *E. coli* RNase E NTD, at a single site, to generate an unlabelled octamer and a 3′ FAM-labelled pentamer product. Inhibitory activity of a small molecule manifests as an increase in the amount of full-length 5′-p-RNA13-FAM-3′ RNA substrate and a decrease in the amount of 3′ FAM-labelled pentamer product detectable at the end of the assay relative to a control assay performed in the absence of small molecule. The inhibitory activity of 0.625, 1.25, 2.5, 5 or 10 mM AS1-9, 5′S1 and 5′S2 was evaluated using this assay (Supplementary Fig. S2). The data obtained in the absence of small molecule and in the presence of 10 mM AS1-4, AS6-9 or 5′S1 are presented in Fig. 2A. At 10 mM, the highest concentration tested, small molecules AS1, AS3 and AS7-9 did not inhibit *E. coli* RNase E NTD. Small molecules AS5 and 5′S2 interfered with the PAGE assay, masking the band representing the 3′ FAM-labelled pentamer product (Supplementary Figs. S2E and K). However, no full-length 5′-p-RNA13-FAM-3′ RNA substrate could be detected at the end of the assay in the presence of either 10 mM AS5 or 10 mM 5′S2, indicating that they also do not inhibit *E. coli* RNase E NTD. In contrast, 10 mM AS2, AS4 or 5′S1 appeared to almost completely inhibit *E. coli* RNase E NTD and a fourth small molecule, AS6, partially inhibited *E. coli* RNase E NTD. Since AS6, appears to be less effective as an RNase E inhibitor, only inhibitory small molecules AS2, AS4 and 5′S1 were taken forwards for further analysis. The chemical structures of inhibitory small molecules AS2, AS4 and 5′S1 are shown in Fig. 2B with further information presented in Supplementary Table S1.

### 3.3 Characterisation of AS2, AS4 and 5′S1 as small molecule inhibitors of RNase E

**Fig. 3:**
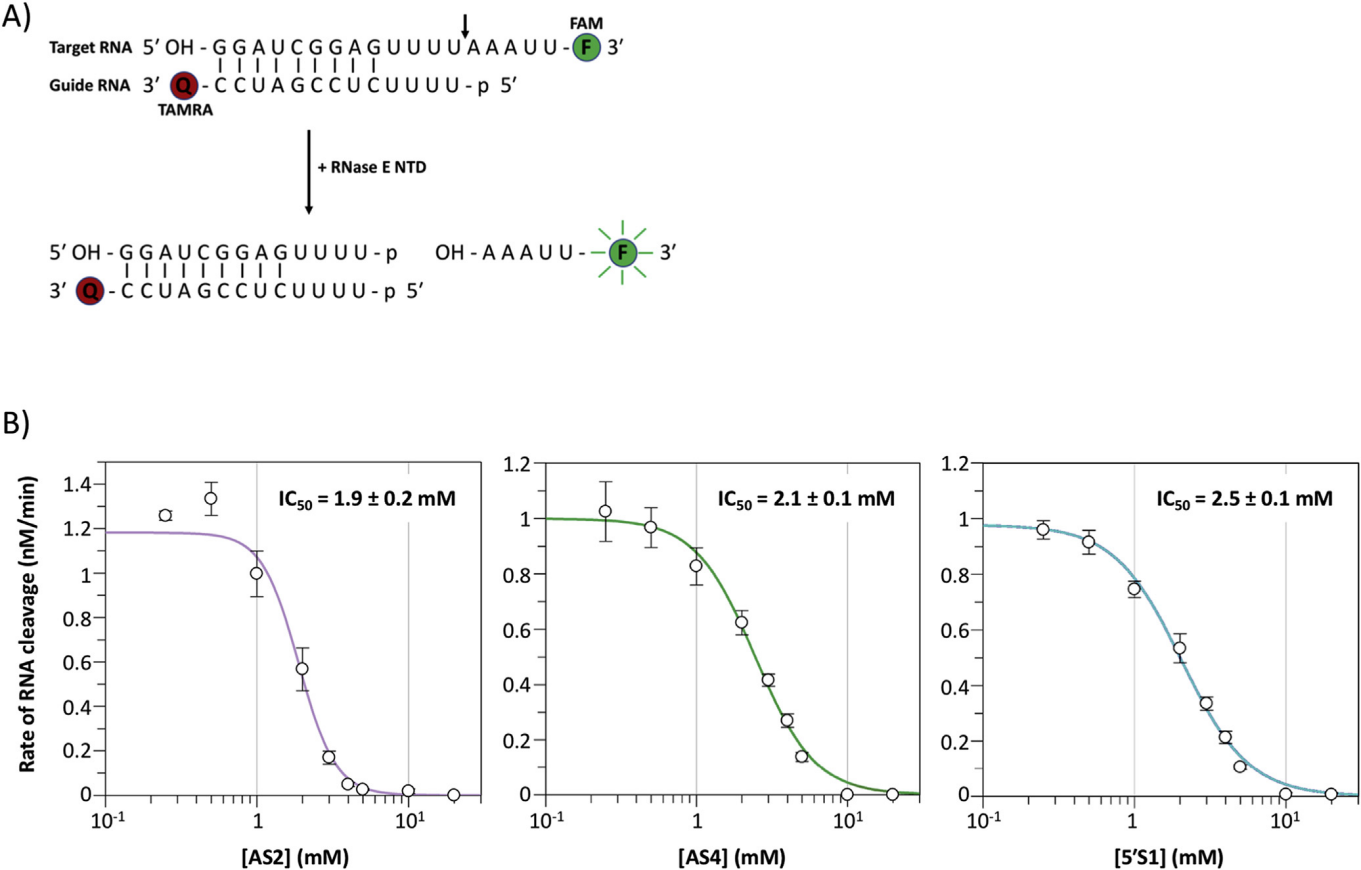
**Potency of AS2, AS4 and 5′S1 as small molecule inhibitors of RNase E.** (A) Schematic of the FRET-based real-time RNase E assay. The substrate is a partially double-stranded RNA (modified target-guide) consisting of a 5′ hydroxylated, 3′ FAM-labelled 18-mer “target” RNA and a 5′ monophosphorylated, 3′ TAMRA-labelled 13-mer “guide” RNA, which anneals to the target RNA with partial complementarity. In the uncleaved modified target-guide substrate, the fluorescence of the 3′ FAM group of the 18-mer target RNA is quenched by the 3′ TAMRA group of the 13-mer guide RNA. Endoribonucleolytic cleavage of the single-stranded A/U-rich region of the 18-mer target RNA by RNase E NTD, at the position indicated by the arrow, results in the release of a 3′ FAM-labelled pentamer and the unquenching of the FAM fluorescence. The increase in fluorescence can be monitored in real-time. (B) Plots of the rate of cleavage of 1 μM modified target-guide RNA substrate by 5 nM E*. coli* RNase E NTD in the presence of 0.25, 0.5, 1, 2, 3, 4, 5 and 10 mM AS2, AS4 or 5′S1. Data are the average from three experiments and the error bars represent the standard error of the mean. Data were fitted as described in Materials and Methods to determine the IC_50_, which is indicated for the respective small molecule inhibitor in the top right-hand corner of the plot.

To further characterise the inhibitory activity of AS2, AS4 and 5′S1 against RNase E, we developed a novel fluorescence resonance energy transfer (FRET)-based real-time RNase E assay. We first designed a partially double-stranded substrate consisting of a 5′ hydroxylated, 3′ FAM-labelled 18-mer “target” RNA and a 5′ monophosphorylated, 3′ TAMRA-labelled 13-mer “guide” RNA, which anneals to the target RNA with partial complementarity (Fig. 3A). This substrate is based on a model target-guide substrate [32]. The 5′ hydroxyl of the target RNA strand prevents direct binding and cleavage of this strand by RNase E [32]. However, the 5′ monophosphate of the guide RNA facilitates RNase E-binding and promotes cleavage of the single-stranded A/U-rich region of the adjacent target RNA strand [32]. In the uncleaved substrate (modified target-guide), the fluorescence of the 3′ FAM group of the target RNA is quenched by the 3′ TAMRA group of the guide RNA (Fig. 3A). Endoribonucleolytic cleavage of the 18-mer target RNA by RNase E NTD results in the release of a 3’ FAM-labelled pentamer and unquenching of the FAM fluorescence (Fig. 3A). This can be monitored in real-time as an increase in fluorescence over time.

We decided to use the FRET-based real-time RNase E assay to investigate the dose-dependent inhibition of RNase E by AS2, AS4 and 5′S1. Each of these three inhibitors had appeared to display dose-dependent inhibition of *E. coli* RNase E NTD in the discontinuous assay (see Supplementary Fig. S2B, D and J). To quantify this, the FRET-based real-time RNase E assay was used to measure the rate of cleavage of 1 μM of the modified target-guide RNA substrate by 5 nM *E. coli* RNase E NTD in the presence of 0.25, 0.5, 1, 2, 3, 4, 5 and 10 mM AS2, AS4 or 5′S1. These data were then used to calculate the IC_50_ for each of the small molecules, as described in Materials and Methods. IC_50_ values of 1.9 mM, 2.1 mM and 2.5 mM were calculated for AS2, AS4 and 5′S1, respectively (Fig. 3B).

Next we used the FRET-based real-time RNase E assay to compare the kinetics of cleavage of the modified target-guide substrate by *E. coli* RNase E NTD. The rate of cleavage of 0.1, 0.2, 0.25, 0.3, 0.35, 0.4, 0.45, 0.5, 0.55, 0.6, 0.8, 1 and 2 μM of the modified target-guide RNA substrate by 5 nM *E. coli* RNase E NTD in the absence of small molecule inhibitors and in the presence of 2 mM AS2, AS4 or 5′S1 was measured. An inhibitor concentration of 2 mM was chosen because this was approximately equal to the calculated IC_50_ for all three small molecule inhibitors (see Fig. 3B). As described in Materials and Methods, these data were then used to calculate the kinetic parameters, V_max_ and K_m_, which are presented in Table 1. The presence of any one of the three small molecule inhibitors both reduced the rate of cleavage of the modified target-guide RNA substrate by *E. coli* RNase E NTD (decreased V_max_) and increased the affinity between *E. coli* RNase E NTD and the modified target-guide RNA substrate (decreased K_m_). This form of modulation often occurs when an effector/inhibitor binds at a site close to, but distinct from, the substrate binding pocket and changes the conformation of the enzyme to favour substrate binding, which also reduces the enzyme catalytic rate. Therefore, AS2, AS4 and 5′S1 appear to be allosteric inhibitors of RNase E.

**Table 1:** **Kinetic parameters for cleavage of the modified target-guide substrate by *E. coli* RNase E NTD.** The rate of cleavage of 0.1, 0.2, 0.25, 0.3, 0.35, 0.4, 0.45, 0.5, 0.55, 0.6, 0.8, 1 and 2 μM of the modified target-guide RNA substrate by 5 nM *E. coli* RNase E NTD in the absence of small molecule inhibitors and in the presence of 2 mM AS2, AS4 or 5′S1 was measured. Each experiment was performed in triplicate. Data were fitted as described in Materials and Methods to determine the kinetic parameters, V_max_ and K_m_.

Inhibitor	V_max_ (pmole/s)	K_m_ (nM)
None	0.29 ± 0.01	268 ± 35
AS2	0.12 ± 0.01	64 ± 41
AS4	0.16 ± 0.02	96 ± 48
5′S1	0.15 ± 0.01	100 ± 26

### 3.4 Inhibition of *F. tularensis* and *A. baumannii* RNase E NTDs by AS2, AS4 and 5′S1

As small molecule inhibitors of RNase E, AS2, AS4 and 5′S1 are potential lead molecules for the development of antibacterial agents targeting RNase E. For an antibiotic to be used effectively, it is useful to know which bacterial species it is active against. Therefore, we decided to screen AS2, AS4 and 5′S1 against RNase E NTDs from bacterial species of importance to the health and/or defence sectors. We selected the pathogens *A. baumannii* and *F. tularensis* because they met this criteria [21,22], and we have previously characterised the structural and biochemical properties of their RNase E NTDs [28].

**Fig. 4:**
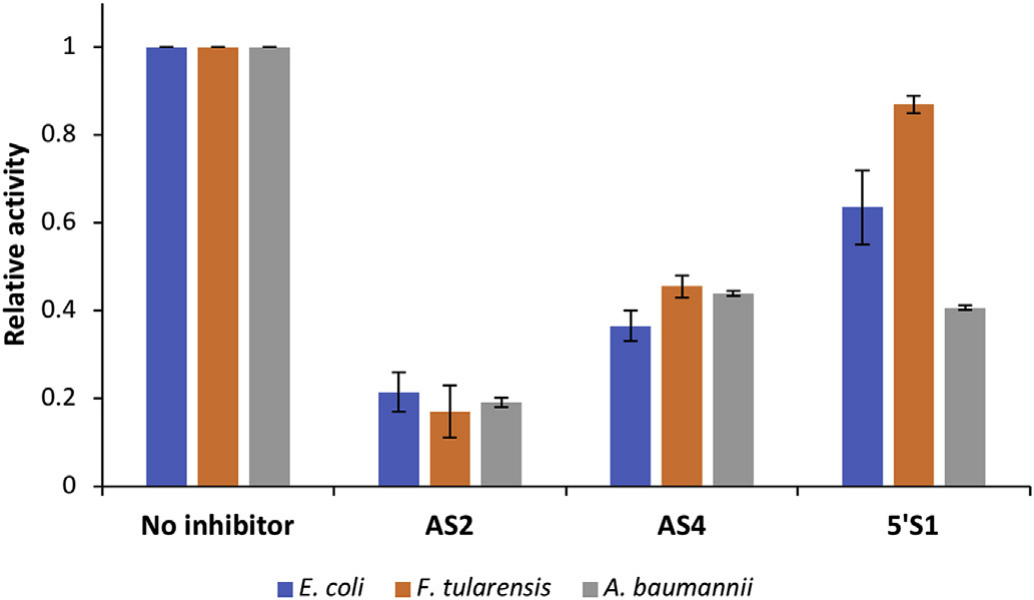
Inhibition of *F. tularensis* and *A. baumannii* RNase E NTDs by AS2, AS4 and 5′S1. The relative rates of cleavage of 1 μM modified target-guide substrate by 5 nM *E. coli* (blue), *F. tularensis* (orange) or *A. baumannii* (grey) RNase E NTD in the absence of inhibitor and in the presence of either 2 mM AS2, AS4 or 5′S1. For each RNase E NTD, the data have been normalised to the cleavage rate when there was no inhibitor present. Data are the average from duplicate experiments and the error bars represent the standard error of the mean. . (For interpretation of the references to colour in this figure legend, the reader is referred to the Web version of this article.)

Using the FRET-based real-time RNase E assay, we screened AS2, AS4 and 5′S1 against *F. tularensis* and *A. baumannii* RNase E NTDs. The rate of cleavage of 1 μM modified target-guide substrate by 5 nM E*. coli*, *F. tularensis* or *A. baumannii* RNase E NTD was determined in the absence of small molecule inhibitors and in the presence of either 2 mM AS2, AS4 or 5′S1 (Fig. 4). All three small molecules inhibited all three RNase E NTDs. This was expected given that the key amino acids at the RNase E active site and the 5′ sensor region, the sites where the small molecule inhibitors are predicted to bind/block, are absolutely conserved between *E. coli*, *F. tularensis* and *A. baumannii* [28]. Furthermore, AS2 and AS4 had a similar effect on the activity of the RNase E NTD from all three species. However, there was more variability in the relative inhibitory activity of 5′S1 between the RNase E NTDs. Approximately 60% inhibition was observed with *A. baumannii* RNase E NTD while *E. coli* RNase E NTD was inhibited approximately 40% and *F. tularensis* RNase E NTD was only inhibited by around 10%. These differences would not have been predicted based on the conservation of key amino acids at the 5′ sensor region between these three species [28]. The significant difference with *F. tularensis* RNase E NTD is interesting given that it has also been reported to exhibit marked differences in substrate cleavage-site specificity compared to the same RNase E NTD homologues [28].

## 4 Discussion

Using a combination of structure-based vHTS and *in vitro* activity screening, we have identified three novel small molecule inhibitors of the potential antibacterial target RNase E: AS2, AS4 and 5′S1 (Fig. 1, Fig. 2). Structure-based vHTS has previously been validated as a suitable approach for identifying effective small molecule inhibitors of RNase E [20]. However, in contrast to the inhibitors identified in the Kime et al. study [20], the three inhibitors identified here are commercially available and inexpensive (costing less than £2.50 per mg). This was achieved by starting with a screening library of commercially available small molecules and then applying a monetary cutoff filter to the vHTS results. Although we attempted to mask small molecules with known desirable bioactivity from the monetary cutoff filter, we acknowledge that by using cost as a filter we may have inadvertently discarded promising inhibitors. Nevertheless, using this approach, we have successfully identified the first universally accessible small molecule inhibitors of RNase E.

With regard to their inhibitory properties, AS2, AS4 and 5′S1 are comparable to the small molecule inhibitors reported in Kime et al. [20]. In each case, the IC_50_ is in the low millimolar range (Fig. 3) and there is evidence that they could all have a broad-spectrum effect (Fig. 4). As potential lead molecules in the development of an antibacterial strategy targeting RNase E, next steps may include exploring the chemical elaboration of the small molecule inhibitors, in an attempt to improve their IC_50_s, and understanding the molecular details of their mode-of-action. Since all three small molecule inhibitors were derived from a screening library of chemical building blocks, chemical modification should be possible in each case. Understanding their mode-of-action is much more challenging. Although AS2, AS4 and 5′S1 can be docked into the active site or 5′ sensor region of RNase E, the RNase E-docked small molecule complex conformations are only predictions and it is far from certain how, or where, the small molecule inhibitors actually bind. Critically, our kinetics data suggest allosteric inhibition of RNase E by all three small molecule inhibitors, implying that they bind distal to the active site, and the effect of 5′S1 on RNase E NTDs from different species is variable, despite the absolute conservation of key amino acids in this region. This raises questions about the assumed binding sites of the small molecule inhibitors. Elucidating the molecular details of the binding interactions and mode-of-action for each of the inhibitors will likely require high-resolution structural studies combined with mutational and molecular dynamics approaches.

Intriguingly, one of the identified small molecule inhibitors of RNase E, AS4, is GlucN6P, a metabolite that is a precursor of bacterial cell envelope peptidoglycans and lipopolysaccharides in the hexosamine pathway (reviewed in Ref. [33]). The production of GlcN6P from l-glutamine and fructose-6-phosphate by GlcN6P synthase (GlmS) is the first step in the synthesis of the bacterial cell envelope and GlcN6P homeostasis, is tightly controlled through feedback inhibition loops that modulate translation of GlmS [33]. In Gram-negative bacteria, GlmS levels are modulated through a regulatory circuit involving RNase E-mediated cleavage of *glmS*-stabilising sRNA GlmZ (reviewed in Ref. [34]). In the presence of high intracellular concentrations of GlcN6P, the adaptor protein RapZ specifically interacts with RNase E and GlmZ, targeting GlmZ for degradation by RNase E [34]. Exactly how direct inhibition of RNase E by GlcN6P might fit into these mechanisms remains to be determined. However, it could serve as a fine-tuning function in the regulation GlcN6P synthesis. Given that the intracellular concentration of GlucN6P in *E. coli* is 1.15 mM when glucose is used as the carbon source [35] and we have determined the IC_50_ of AS4/GlucN6P to be 2.1 mM *in vitro* (Fig. 3), it is theoretically possible that GlucN6P could regulate RNase E *in vivo.* Such regulation would provide support for a communicative link between RNases and cellular metabolic status that has been proposed previously [36,37].

## 5 Conclusion

In summary, we have demonstrated inhibition of RNase E NTD by three novel small molecule inhibitors, AS2, AS4/GlucN6P and 5′S1, *in vitro*. These small molecules have potential as lead molecules for the development of an antibacterial strategy targeting RNase E. In addition, AS4/GlucN6P, as a natural metabolite, is a potential regulator of RNase E. To the best of our knowledge, this is the first time that RNase E activity has been shown to be directly modulated by a small molecule metabolite.

## CRediT author statement

**Charlotte E. Mardle:** Conceptualization, Methodology, Investigation, Formal analysis, Writing – Original Draft, Writing – Review & Editing; **Layla R. Goddard:** Investigation, Formal analysis, Writing – Review & Editing; **Bailei C. Spelman:** Investigation, Writing – Review & Editing; **Helen S. Atkins:** Conceptualization, Writing – Review & Editing; **Louise E. Butt:** Conceptualization, Methodology, Writing – Review & Editing, Supervision; **Paul A. Cox:** Conceptualization, Methodology, Writing – Review & Editing; **Darren M. Gowers:** Conceptualization, Methodology, Formal analysis, Writing – Original Draft, Writing – Review & Editing; **Helen A. Vincent:** Conceptualization, Formal analysis, Writing – Original Draft, Writing – Review & Editing; **Anastasia J. Callaghan:** Conceptualization, Methodology, Formal analysis, Writing – Original Draft, Writing – Review & Editing, Supervision, Funding acquisition.

## Declaration of competing interest

Dr Mardle, Miss Goddard, Mr Spelman, Dr Butt, Dr Cox, Dr Gowers, Dr Vincent and Prof Callaghan report grants from 10.13039/100010418Defence Science and Technology Laboratory and grants from 10.13039/501100000268Biotechnology and Biological Sciences Research Council, during the conduct of the study.

Dr Atkins reports grants from 10.13039/100010418Defence Science and Technology Laboratory, during the conduct of the study. Dr Atkins was an employee of Defence Science and Technology Laboratory, one of the co-funders of the research, during the conduct of the study.

## Abbreviations

DMSO: dimethyl sulfoxide
DTT: dithiothreitol
EDTA: ethylenediaminetetraacetic acid
FAM: fluorescein amidite
FRET: fluorescence resonance energy transfer
GlmS: GlucN6P synthase
GlucN6P: glucosamine-6-phosphate
IC_50_: half maximal inhibitory concentration
IPTG: isopropyl β-d-1-thiogalactopyranoside
MOE: Molecular Operating Environment
NTD: N-terminal domain
PAGE: polyacrylamide gel electrophoresis
RMSD: root-mean-square deviation
RNase(s): ribonuclease(s)
TAMRA: tetramethyl-6-carboxyrhodamine
vHTS: virtual high-throughput screening
