# Supplementary material for: Identification and analysis of novel small molecule inhibitors of RNase E: Implications for antibacterial targeting and regulation of RNase E
Source: Biochem Biophys Rep. 2020 Jun 9;23:100773. doi: 10.1016/j.bbrep.2020.100773 (PMC7284133; doi:10.1016/j.bbrep.2020.100773)
Supplement: Multimedia component 1 [file mmc1.pdf]

Appendix A. Supplementary data

Identification and analysis of novel small molecule inhibitors of RNase E: Implications for antibacterial targeting and regulation of RNase E

Charlotte E. Mardle[a], Layla R. Goddard[a], Bailei C. Spelman[a], Helen S. Atkins[b,c,d], Louise E. Butt[a], Paul A. Cox[e], Darren M. Gowers[a], Helen A. Vincent[a,]** and Anastasia J. Callaghan[a,]*

[a] School of Biological Sciences and Institute of Biological and Biomedical Sciences, University of Portsmouth, Portsmouth, PO1 2DY, United Kingdom

[b] Defence Science and Technology Laboratory, Porton Down, Salisbury, United Kingdom

[c] University of Exeter, Exeter, United Kingdom

[d] London School of Hygiene and Tropical Medicine, London, United Kingdom

[e] School of Pharmacy and Biomedical Sciences and Institute of Biological and Biomedical Sciences, University of Portsmouth, Portsmouth, PO1 2DT, United Kingdom

* Corresponding author.

** Corresponding author.

Email addresses: Helen.Vincent@port.ac.uk (H.A. Vincent); Anastasia.Callaghan@port.ac.uk (A.J. Callaghan)

References

[1] Callaghan,A.J., Marcaida,M.J., Stead,J.A., McDowall,K.J., Scott,W.G. & Luisi,B.F. (2005) Structure of *Escherichia coli* RNase E catalytic domain and implications for RNA turnover. *Nature* **437**, 1187-1191.

[2] Chao,Y., Li,L., Girodat,D., Förstner,K.U., Said,N., Corcoran,C., Śmiga,M., Papenfort,K., Reinhardt,R., Wieden,H.J., Luisi,B.F. & Vogel,J. (2017) *In vivo* cleavage map illustrates the central role of RNase E in coding and non-coding RNA pathways. *Mol. Cell* **65**, 39-51.

[3] Bandyra,K.J., Wandzik,J.M. & Luisi,B.F. Substrate recognition and autoinhibition in the central ribonuclease RNase E. *Mol. Cell* **72**, 275-285.e4 (2018).

Supplementary Table S1. Candidate small molecule inhibitors selected for *in vitro* screening

Compound code/name	ZINC ID/ PubChem CID	Target binding site in RNase E	Docking score/ S value (kcal/mol)	Supplier/ Cost	2D structure
AS1/ 1,2-Bis(2-aminophenoxy) ethane-*N,N,N′,N′*-tetraacetic acid	3860554/ 104751	Active site	-124.8	Sigma/ £0.13 per mg	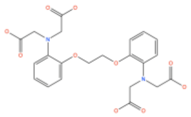
AS2/ 3-[(4-Hydroxy-5-isopropyl-6-oxo-1,6-dihydro-2-pyrimidinyl) sulfanyl]propanoic acid	19735178/ 329779174	Active site	-104.8	Sigma/ £1.26 per mg	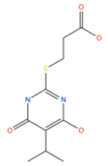
AS3/ Heparin	34888464/ 16219490	Active site	-98.8	Sigma/ £91.50 per mg	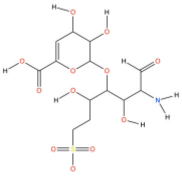
AS4/ D-Glucosamine-6-phosphate	04097102/ 440997	Active site	-75.7	Sigma/ £2.48 per mg	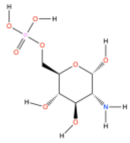
AS5/ Kasugamycin	4216682/ 65174	Active site	-58.8	Sigma/ £0.03 per mg	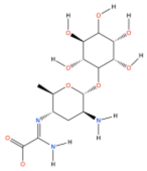
AS6/ 4-O-β-Galactopyranosyl-D-mannopyranoside (Epilactose)	26892042/ 4195243	Active site	-57.5	Sigma/ £4.44 per mg	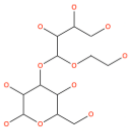
AS7/ Antazoline	57204/ 2200	Active site	-36.9	Sigma/ £0.01 per mg	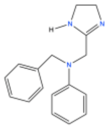
AS8/ Phentolamine	20251/5775	Active site	-28.0	Sigma/ £0.66 per mg	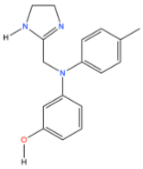

Compound code/name	ZINC ID/ PubChem CID	Target binding site in RNase E	Docking score/ S value (kcal/mol)	Supplier/ Cost	2D structure
AS9/ Oxymetazoline	57435/4636	Active site	-24.2	Sigma/ £0.02 per mg	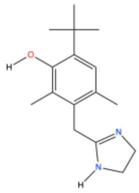
5′S1/ 3-(3-Methyl-5-oxo-4,5-dihydro-1H-pyrazol-4-yl) propanoic acid	18082626/ 2771950	5′ sensor	-22.8	Sigma/ £0.63 per mg	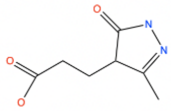
5′S2/ 2-(5-sulfanyl-4H-1,2,4-triazol-3-yl) phenol	16957372/ 5388408	5′ sensor	-21.7	Sigma/ £1.26 per mg	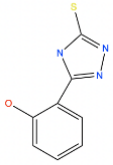

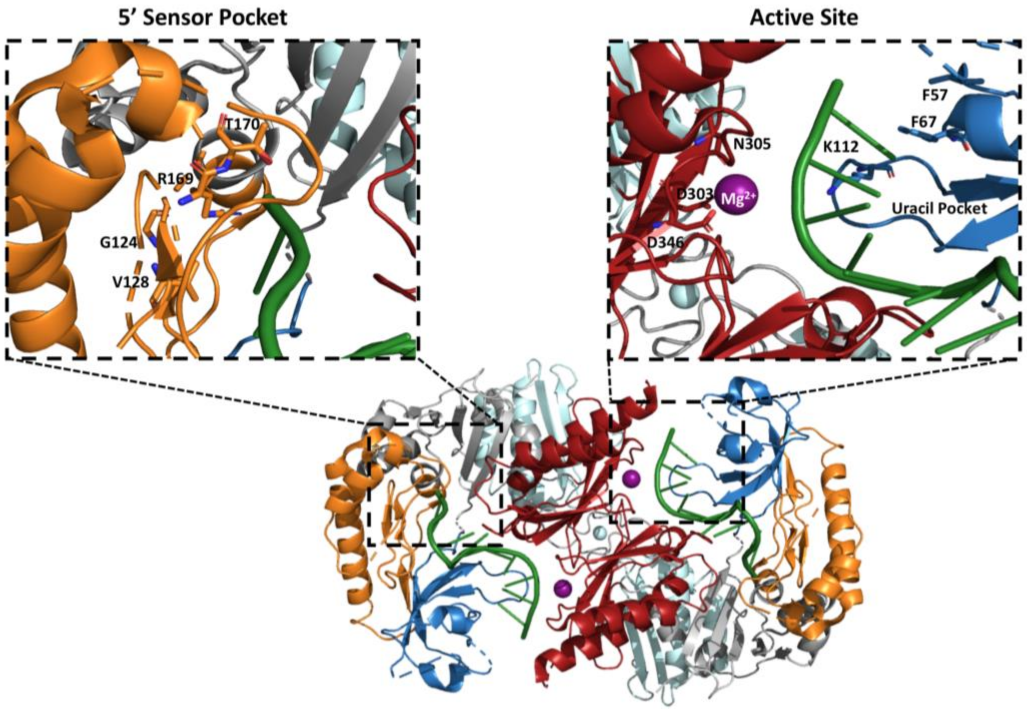

Supplementary Fig. S1. Structure of *E. coli* RNase E NTD. *E. coli* RNase E NTD dimer (PDB accession code: 2C0B; [1]) shown as ribbons and coloured by subdomain: RNase H domain (grey), S1 domain (blue), 5′ sensor domain (gold), DNase I domain (red) and small domain (cyan). Mg^{2+} ions are shown as magenta spheres and Zn^{2+} ions as cyan spheres. The RNA substrate is shown in green. The active site and 5′ sensor region are both enlarged in the zoom-in panels with key amino acids, known to be critical for substrate recognition and/or cleavage, shown as labelled sticks [1-3]. The active site contains an essential catalytic magnesium ion coordinated by two aspartates (D303, positioned by N305, and D346) from the DNase I subdomain and the RNA-binding uracil pocket (including key amino acids F57, F67 and K112) from the S1 subdomain [1,2]. The phosphorylation state of the substrate is recognised by the 5′ sensor region through interactions between a 5′ monophosphate on an RNA substrate and a conserved arginine and threonine (R169 and T170) from the 5′ sensor subdomain [1-3].

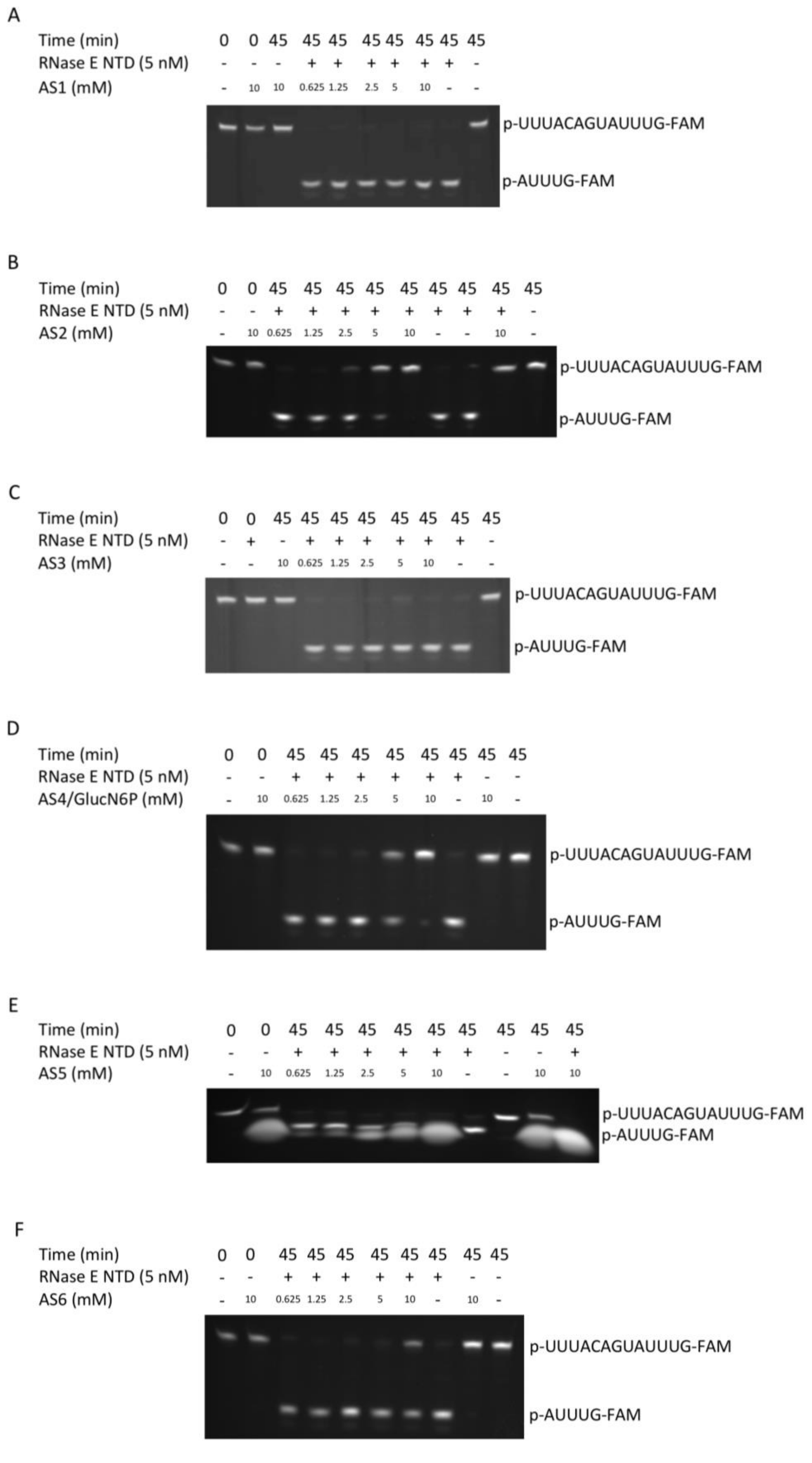
A
Time (min) 0 0 45 45 45 45 45 45 45 45
RNase E NTD (5 nM) - - - + + + + + + -
AS1 (mM) - 10 10 0.625 1.25 2.5 5 10 - -
p-UUUACAGUAUUUG-FAM
p-AUUUG-FAM
B
Time (min) 0 0 45 45 45 45 45 45 45 45 45
RNase E NTD (5 nM) - - + + + + + + + + -
AS2 (mM) - 10 0.625 1.25 2.5 5 10 - - 10 -
p-UUUACAGUAUUUG-FAM
p-AUUUG-FAM
C
Time (min) 0 0 45 45 45 45 45 45 45 45
RNase E NTD (5 nM) - + - + + + + + + -
AS3 (mM) - - 10 0.625 1.25 2.5 5 10 - -
p-UUUACAGUAUUUG-FAM
p-AUUUG-FAM
D
Time (min) 0 0 45 45 45 45 45 45 45 45
RNase E NTD (5 nM) - - + + + + + + - -
AS4/GlucN6P (mM) - 10 0.625 1.25 2.5 5 10 - 10 -
p-UUUACAGUAUUUG-FAM
p-AUUUG-FAM
E
Time (min) 0 0 45 45 45 45 45 45 45 45 45
RNase E NTD (5 nM) - - + + + + + + - - +
AS5 (mM) - 10 0.625 1.25 2.5 5 10 - - 10 10
p-UUUACAGUAUUUG-FAM
p-AUUUG-FAM
F
Time (min) 0 0 45 45 45 45 45 45 45 45
RNase E NTD (5 nM) - - + + + + + + - -
AS6 (mM) - 10 0.625 1.25 2.5 5 10 - 10 -
p-UUUACAGUAUUUG-FAM
p-AUUUG-FAM

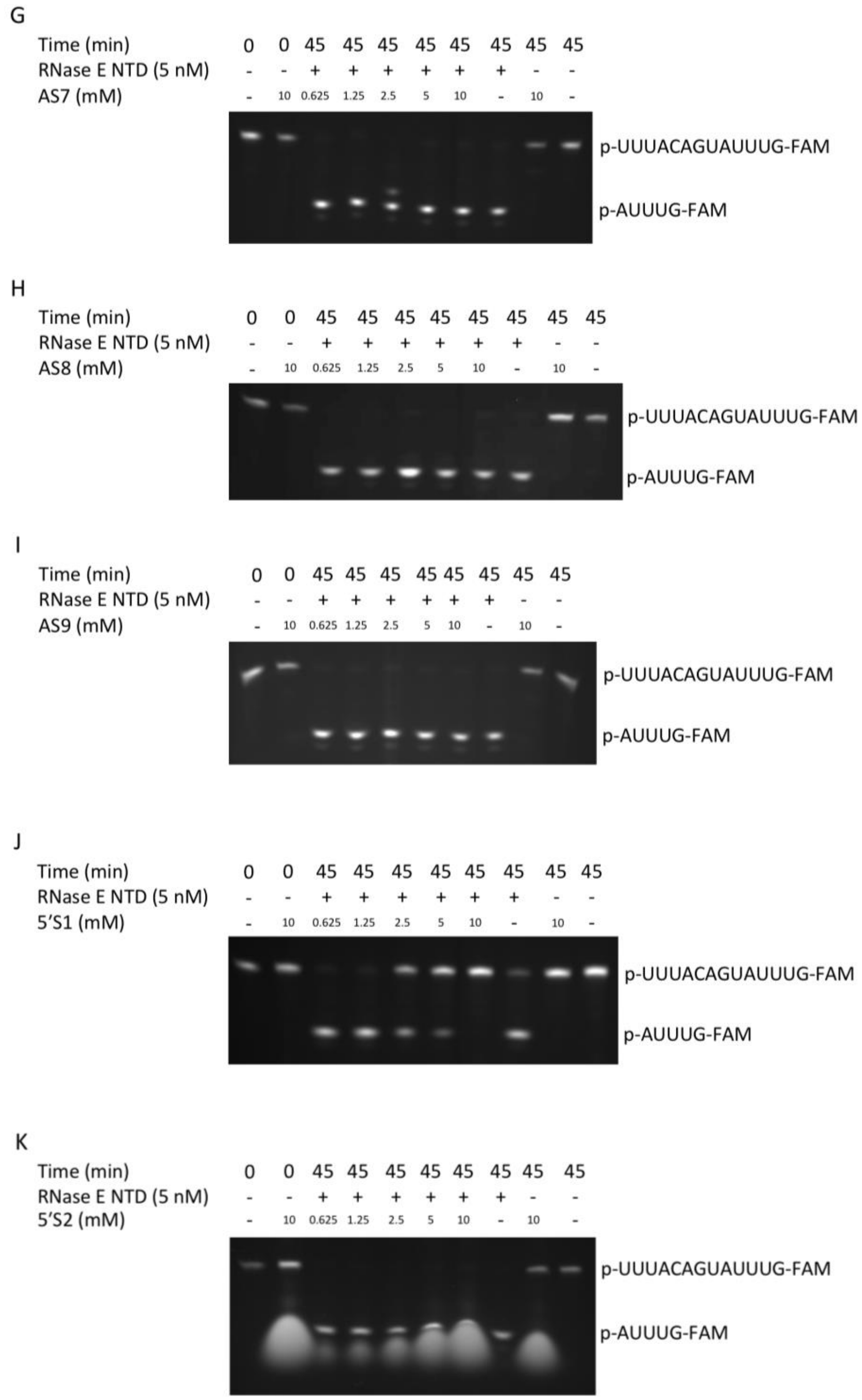

Supplementary Fig. S2. ***In vitro* screening of candidate small molecule inhibitors of RNase E against *E. coli* RNase E NTD.** Representative 20% denaturing PAGE analysis of the cleavage of 1 μM 5′-p-RNA13-FAM-3′ by 5 nM *E. coli* RNase E NTD after incubation at 28°C for 45 min in the absence of small molecule (-) or in the presence of 0.625, 1.25, 2.5, 5 or 10 mM candidate small molecule

inhibitor, as indicated above the lanes. The expected positions of the bands representing the full-length FAM-labelled 5′-p-RNA13-FAM-3′ substrate and the FAM-labelled pentamer 5′-p-AUUUG-FAM-3′ cleavage product are indicated on the right hand side of the gels. A) AS1, B) AS2, C) AS3, D) AS4, E) AS5, F) AS6, G) AS7, H) AS8, I) AS9, J) 5′S1 and K) 5′S2.
